# Timely Reporting and Interactive Visualization of Animal Health and Slaughterhouse Surveillance Data in Switzerland

**DOI:** 10.3389/fvets.2015.00047

**Published:** 2015-10-29

**Authors:** Ulrich J. Muellner, Flavie Vial, Franziska Wohlfender, Daniela Hadorn, Martin Reist, Petra Muellner

**Affiliations:** ^1^Veterinary Public Health Institute, Department of Clinical Research and Veterinary Public Health, Vetsuisse Faculty, University of Bern, Bern, Switzerland; ^2^Epi-interactive, Wellington, New Zealand; ^3^Epi-interactive, Eppingen, Germany; ^4^Federal Food Safety and Veterinary Office, Bern, Switzerland; ^5^SAFOSO Inc., Liebefeld, Switzerland

**Keywords:** customizable dashboards, data exploration, early-warning system, information dissemination, open tools, outbreak detection, web application

## Abstract

The reporting of outputs from health surveillance systems should be done in a near real-time and interactive manner in order to provide decision makers with powerful means to identify, assess, and manage health hazards as early and efficiently as possible. While this is currently rarely the case in veterinary public health surveillance, reporting tools do exist for the visual exploration and interactive interrogation of health data. In this work, we used tools freely available from the Google Maps and Charts library to develop a web application reporting health-related data derived from slaughterhouse surveillance and from a newly established web-based equine surveillance system in Switzerland. Both sets of tools allowed entry-level usage without or with minimal programing skills while being flexible enough to cater for more complex scenarios for users with greater programing skills. In particular, interfaces linking statistical softwares and Google tools provide additional analytical functionality (such as algorithms for the detection of unusually high case occurrences) for inclusion in the reporting process. We show that such powerful approaches could improve timely dissemination and communication of technical information to decision makers and other stakeholders and could foster the early-warning capacity of animal health surveillance systems.

## Introduction

Early detection of disease outbreaks or changes in the frequency of disease in populations plays a vital role in reducing the impact of both emerging and endemic zoonotic diseases (1, 2). To support interpretation of surveillance data by relevant stakeholders, visualization of health surveillance data (e.g., mapping and charting) is commonly done to show changes or patterns in the data, such as an increase in disease incidence over a specific time period at the national level or in a specific geographic region or population group (3, 4). While early detection of aberrations in surveillance data provides a powerful means to identify, assess, and respond to events, reporting of animal health surveillance data is most often neither real-time (or near real-time) nor interactive, two attributes, which would greatly enhance the ability of the surveillance system to meet its objectives. In addition, visual and interactive approaches to reporting can facilitate evidence-based decision making by risk managers. Such an approach permits the interrogation and exploration of the data by the user, which in return allows for a much better understanding of the inherent complexity that animal and human health data naturally entail.

Google Maps and Charts (5, 6) are widely available multi-device-capable software tools for the visualization of data. The value of Google Maps in the context of public health has previously been described (7). The interactive web-based charts and maps that can be built from those tools provide an easily accessible means to better display surveillance data to provide information that can readily be translated into public health, animal health, and food safety actions.

The objective of the work presented was to develop the proof of concept for a web application capable of displaying different types of Swiss animal health surveillance data to improve early detection of outbreaks and changes in the health status of the country’s animal population by the Swiss Federal Food Safety and Veterinary Office (FSVO). For this purpose, data from post-mortem meat inspection and from a newly established web-based surveillance system for equine diseases were utilized.

## Methods and Results

### Post-Mortem Meat Inspection Data

**Figure 1:**
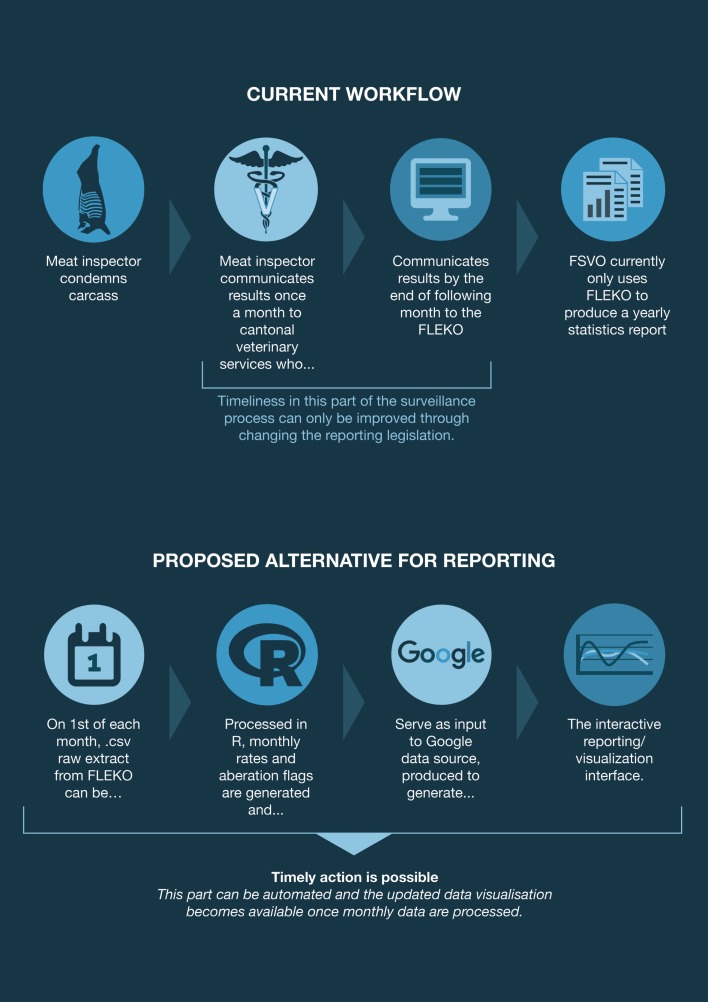
**Swiss post-mortem meat inspection data from collection to visualization**. Current workflow and proposed semi-automated alternative for reporting.

Since 2007, the official veterinarians in charge of meat inspection and the cantonal veterinary services use the “Fleischkontrolldatenbank” (FLEKO federal database) to communicate meat inspection results to the FSVO on a monthly basis. The FLEKO holds post-mortem meat inspection data from all hoofed animals slaughtered in Switzerland. Depending on the observations made by the meat inspector (none, generalized vs. localized conditions) on the carcass, the carcass can either be (1) classified as entirely fit for human consumption; (2) wholly condemned (this includes organs and blood); or (3) partially condemned (only parts of the carcass unfit for human consumption are removed). Meat inspectors report to the FLEKO the number of normal and emergency (sick or injured) slaughtered animals, the number of whole carcass condemnations (WCC), and the reason for WCC. More information on meat inspection in Switzerland and the FLEKO database is described in details elsewhere (8). These data were primarily not collected for surveillance purposes but for economic purposes, namely to calculate the amount of subsidies to be paid to each slaughterhouses for the disposal of animal by-products (amount per animal slaughtered). As such, the data have been underutilized by the FSVO, only appearing in a yearly summary report. We sought to improve its usability for surveillance by devising a timely and semi-automated way to report and visualize key indicators from the data, which can be interpreted by animal health experts on a monthly basis (Figure 1).

### Equine Health Data

**Figure 2:**
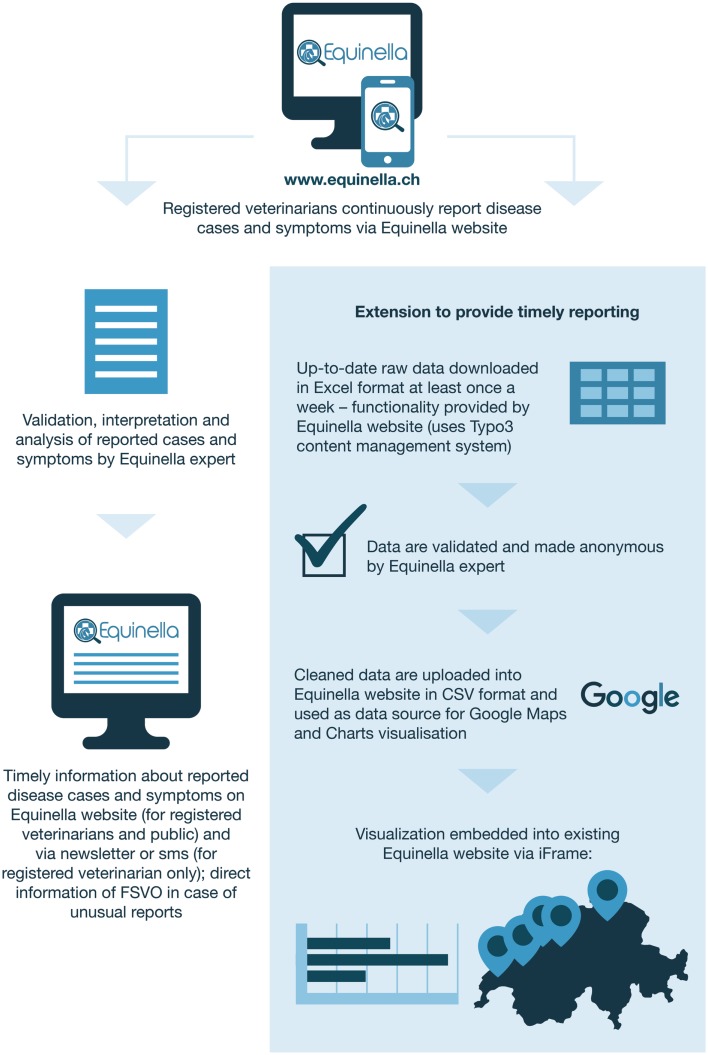
**Swiss equine health data from collection to visualization**. Extension to provide timely reporting.

Equinella^1^ is a voluntary reporting system for equine infectious diseases in Switzerland that was established in 1990. The system’s focus lies on equine diseases that are not notifiable by Swiss law. In 2012, an evaluation of the system showed that it was not representative anymore of the Swiss horse population (9). A survey performed among veterinarians in order to assess the requirements and expectations for an improved reporting system revealed the following: a majority of veterinarians prefer to report cases electronically; at least half of them are willing to report syndromes in addition to disease cases; many are willing to report events on a daily basis (10). Based on the findings from that survey, a new electronic reporting system was developed in 2013 as a collaboration between stakeholders from research (Vetsuisse Faculty, Bern), the Swiss Association of Equine Practitioners, and the FSVO. The system is based on a user-friendly online platform (functional since November 2013) and allows registered veterinary practitioners to report and visualize symptoms as well as cases of equine diseases either using computers or portable devices. Further information on Equinella is described in details elsewhere (9). Unlike FLEKO, Equinella was designed with particular surveillance purposes in mind, and technical solutions for data capture were devised to facilitate a smooth and timely data flow (Figure 2). The continuous near real-time analyses of the Equinella data benefit the early detection of new and (re-)emerging equine diseases; thereby allowing appropriate measures in a timely manner for the protection of the equine population.

### Applications of the Google Maps and Charts library

**Figure 3:**
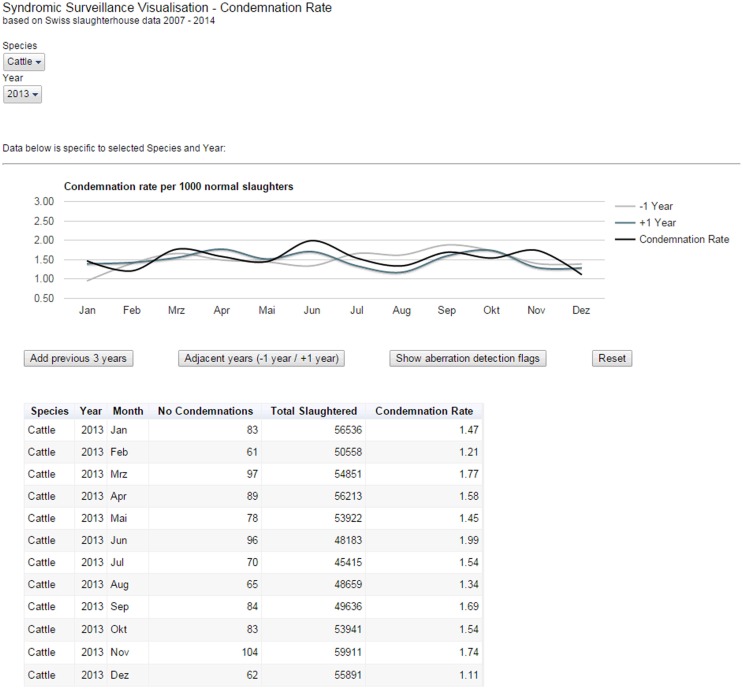
**Reporting dashboard for Swiss post-mortem meat inspection data**. The smoothed bar chart and data table created using Google Charts Tools dynamically display the carcass condemnation rate for a selected species and year(s).

For the post-mortem meat inspection data, Google Charts Tools (5) were used to build an interactive reporting dashboard, which allows using controls to filter data for species, i.e., cattle, pigs, small ruminants, as well as specific years. Depending on the filter chosen, a smoothed bar chart and a data table were displayed, which update dynamically to display the carcass condemnation rate for the selected year and species (Figure 3). The bar chart could be overlaid with either previous or adjunct years. For example, we can see on Figure 3 that the condemnation rate for cattle peaked in June in 2013 (black line) and 2014 (blue line) but that it was not the case in 2012 (gray line). It seems too that the condemnation rate between July and September was higher in 2012 than during the same period in the two following years. While only the output from statistical models can quantitatively define whether the rate at some point in time is significantly above “the norm”; our visualization tool enabled decision makers to qualitatively compare the condemnation rates for adjacent years and to judge whether the rates observed remained within the expectations for the time of year. Furthermore, a provisionary home page was created, which presents the selected data set within the continuum of veterinary surveillance in Switzerland. This is to allow for the expansion of the current system to include additional data sources capable of providing early-warning signals (e.g., laboratory data).

**Figure 4:**
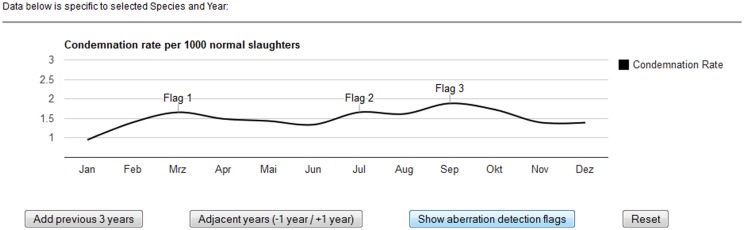
**Displaying statistical aberrations in the post-mortem meat inspection dashboard**. The output of statistical models, determining whether counts at a particular point in time are unusually high, can be displayed dynamically in the chart.

In addition, the Google Charts annotation feature was used to show aberration detection flags. In aberration detection, statistical models determine whether the counts in a given syndrome (in this case WCC) and point in time (in this case month) are unusually high and thus worth investigating further. Aberration detection flags could be added to the underlying data table and the flags displayed dynamically in the chart to highlight certain data points. A quasi-Poisson regression (also known as an improved Farrington) algorithm for the detection of disease outbreaks during post-mortem inspection of slaughtered animals has been positively evaluated on historical FLEKO data (11). It will, in the near future, be fully integrated into the FSVO early-detection system, and statistical aberrations from prospective surveillance data will appear as flags on the visualization interface developed. For the purpose of this project, we used placeholder flags to illustrate the principal functionality (Figure 4).

The reporting dashboard was programed using JavaScript, HTML, and CSS utilizing, in particular, the Controls and Dashboard elements of the Google Charts Tools. This was useful as the tools provided ready-to-use functions to link the various interactive elements of the dashboard, e.g., the filter controls with the chart and data table, while just basic JavaScript code was required to set up the dashboard. The underlying data were added to a Google Fusion Table (12), which was referenced from the JavaScript code. Google Fusion Tables can be managed online with a free Google Docs account, including functions to upload and update data from different sources. Consequently, any change to the underlying Google Fusion data table immediately updates the visualization. The permission to update the Google Fusion Tables can be assigned by the owner to other users, whereby with the free version the view access to the data needs to be set to public. Advanced privacy settings are possible with paid Google subscriptions. However, in our case, data updates were limited to a small group of experts who were familiar with the data updating processes, which did not require more granular access permissions.

**Figure 5:**
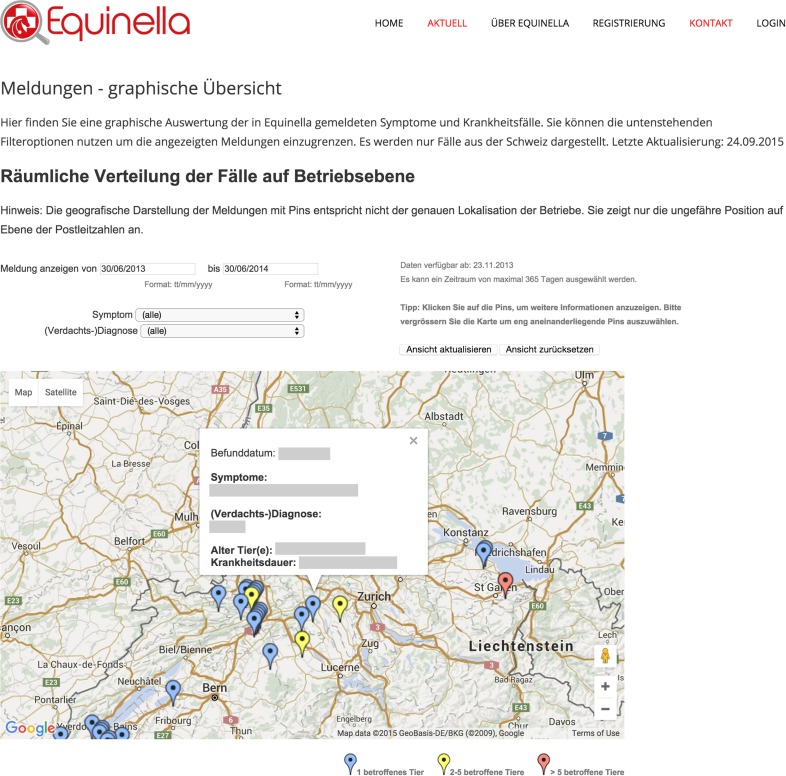
**Interrogative spatial display of Swiss equine health data**. Reported syndromes and diseases in horses are visualized in space and time with the Google Maps API.

**Figure 6:**
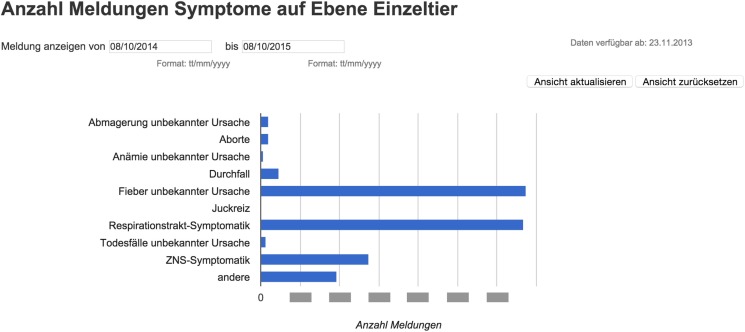
**Reporting dashboard for Swiss equine health data – Google Chart**. The data table can be queried to produce bar charts comparing the frequency of the symptoms and diagnoses reported during a specific time interval.

For the equine dataset, Google Chart Tools were used in combination with the Google Maps application programing interface (API) to visualize reported syndromes and diseases in horses in space and time. The reporting dashboard combined a Google Map displaying reported cases (Figure 5) with charts highlighting the frequency of specific syndromes and diseases. Per default, all cases up to 12 months back were displayed on the map as pins, allowing the user to zoom in and click on pins to access further details. Details displayed per case included the date when the animal was examined, the diagnoses or syndromes reported, as well as the age of the animal, and the duration of illness. In order to report on specific time periods, date filter options were added to manually select a date range. This enabled, for example, a user to just report on cases reported within the last two months. In addition to these temporal filters, filters for symptoms and diseases were available to narrow down the selection and interrogate the data based on the users’ objectives. This included the option to just show cases where symptoms were reported, but no diagnosis could be made at the time of reporting. Underneath the Google Map, bar charts were added to provide a comparison of the frequency of the symptoms and diagnoses reported (Figure 6). Similar to the map view, date filters were added enabling users to select specific data ranges. Because of the limited availability of historical data (the platform has only been functional since November 2013), algorithms for the detection of unusually high case occurrences cannot be parameterized at present, and data interpretation is solely carried out by experts. However, such algorithms can be evaluated and implemented in the near future, as in the FLEKO, based on two or more years of baseline data.

Similar to the first dashboard for post-mortem meat inspection data, the visualization was separated from the data source, using an intermediate table in CSV format as data source. This table was required to support a data validation process, which is manually performed at the very least on a weekly basis, and with a higher frequency when the case load dictates it. At this point also, any data specific to the farm of origin or the owner of the animal were removed from the underlying data due to data protection requirements.

As Google Chart Tools itself just support limited built-in Google Maps features (e.g., no support of HTML info pop-ups), the Google Maps API V3 (6) was used in addition to utilize advanced maps features. Both toolsets were used in combination with JavaScript and jQuery (a JavaScript library designed to simplify native JavaScript scripting) to build the filters. In particular, the Equinella visualization required a greater programing effort as the Google Maps API V3 could not be used in combination with the dashboard features of the Google Chart Tools, which enable easy linking of the filters. This resulted in an approximately three times higher programing effort for the Equinella dashboard (around 1300 lines of code) compared to the FLEKO visualization (around 450 lines of code). Overall, the use of data tables and views provided by the Google Tools was particularly useful for the sorting, filtering, and computing the required data needed for the maps and charts during runtime.

A particular requirement of the map visualization was to show the location of the cases on the level of Swiss postal code level only to protect the privacy of horse owners, veterinarians, and other stakeholders. This was done by using a mapping table, which translated the postal codes of all Swiss cantons into matching latitude/longitude pairs for the visualization (in total over 3500 postal code locations). The mapping table provided a faster and more reliable way to translate the locations than using the built-in Google tools to geocode the locations during runtime. The equine health data visualization was embedded into the existing website of the previously described equine disease surveillance platform, which was built using the Typo 3 content management system^2^.

A recent addition to the map visualization was developed in June 2015 to include outbreak information for a particular location. In order to provide a visual representation, a different color coding was used when either two to five animals (yellow pins) or more than five animals were affected (red pins). Clicking on a yellow or red pin then revealed the exact number of affected animals for a specific date and location.

## Discussion

Google Maps and Google Charts are powerful and easily accessible tools for interactive and near real-time reporting of surveillance data. The use of these or similar tools should be encouraged to support timely dissemination and quick interpretation of surveillance data to improve the use of such data for human and animal health action. Furthermore, these techniques can also support plain reporting of animal health and food safety data, which is increasingly requested by decision makers and other stakeholders to improve dissemination and communication of technical information (13).

Real-time surveillance is a much-desired feature of surveillance as it fosters early-warning capacity. Reducing the lag between data collection and health action is not an easy task and requires a chain of events from the initial sampling procedure, to laboratory testing, database entry, data analysis, and information dissemination to achieve a dynamic process. Automated analysis and display of output is one step toward these goals. Data added to the database instantly become visible, bypassing the need for data extraction and manual analysis by an educated specialist. Expert validation of the data and interpretations to be displayed might still be required, to ensure that the information is fit-for-purpose and correct.

The split of data and presentation layer utilized in this work will also enable building applications that display information and analysis at different levels of permission. For example, the public section of a site may allow access to selected visualization only, while administrators or specific users can access and interrogate all layers of the data. It is crucial that the choice of visualization tools for data interpretation is made in collaboration with an epidemiologist or population health specialist to ensure that reporting standards are met. For example, comparisons made have to be meaningful for the given dataset, relevant, and of value to the situational context. In addition, there is a risk of misinterpretation of the data by less informed end-users. This is not a problem unique to online reporting but of relevance to all reporting of technical information to lay stakeholders. In an online environment, such as the one presented here, careful interface design can help to reduce the risk, for example, by providing information required for the interpretation through context-sensitive interactions, such as explanations of technical terms or interactive guidance and feedback.

The tools described could potentially be used to support regulatory activities, monitoring, and surveillance programs as illustrated by the slaughterhouse data as well as to monitor the health of animal populations as shown here for equines. As a secondary benefit, the equine health approach presented here could support a participatory approach to equine health. Through access to the data on the website, veterinarians and other stakeholders registered in the program gain a sense of community and take increased responsibility for the role they can play to contribute to better animal health.

Privacy and security of data have to be carefully navigated when interacting with Google tools. To meet the requirements of the Swiss Government (e.g., information about animal owners and exact locations of animals could not be displayed), we anonymized the data and used data aggregation methods to avoid that reported cases could be tracked down to individual owners. For example, in the map visualization used, cases were reported at Swiss postcode level (there around 4000 postcodes in Switzerland), and line and bar charts were used to display the data in an aggregated format. All code and data were processed in the browser during runtime. As stated in the Google Charts API Data Policy for the chart types used, no data are stored persistently on any external servers. This was also the case for the maps visualization where the Google Maps API V3 was utilized. As no Google geocoding service was used, no exact location data were processed by Google servers.

Where possible, the development of systems like the one presented is preferable to the time-consuming and error-prone manual creation of graphics, which require on-going extraction of data from the database source, creation of an image-based graph, and then upload into a specific website. As reporting is often a regular requirement, the effort to code real-time, web-based reporting dashboards usually become cost-effective after a short period of time. Resource investment required will soon be offset by reduced effort needed for individual reports and visualizations as well as increased speed of reporting once the system is established.

This work shows that Google tools are by no means a specialist application. They provided a range of toolsets from entry-level usage without or minimal programing skills up to complex usage scenarios. Their user-friendliness allowed practitioners and decision makers to explore the data or to layer information, providing a progression from static, image-based data visualizations to interactive reporting dashboards on which data can be displayed in an epidemiologically meaningful way. Furthermore, these techniques can potentially be extended to include algorithms to provide additional analytical functionality. Such algorithms for Swiss slaughterhouse data have recently been developed (11) and their output will be incorporated into the dashboard in the future.

The use of interactive data visualization tools to foster the dialog between data analysts and others has been popularized by Hans Rosling’s TED presentation in 2006^3^. Google has been a leader in this field ever since, with the Google Charts API allowing users to create interactive charts as part of Google documents, spreadsheets, and web pages since 2007. A testimony of the power of these tools is the recent development of interfaces between Google Charts API and other commonly used analytical software such as the R (14) or SAS (15) softwares. R has become the most popular language for data science and millions of data scientists use R to solve challenging quantitative problems in fields ranging from computational biology to finances. For example, the surveillance package (16) contains implementation of statistical methods for the modeling and change-point detection in time series of counts, as well as for the modeling of continuous-time epidemic phenomena, which was used on the slaughterhouse surveillance dataset (11). The googleVis package (17) provides an interface between R and the Google Charts API, allowing the user to visualize data stored in R data frames with Google Charts without uploading the data to Google (which we did to produce the interface for FLEKO presented above). The output of a googleViz function is HTML code with the data embedded and can be added to any website. The downside is that the data are embedded in a static way and extensive R coding knowledge is required to build or change the visualization making it less accessible than the Google Charts API.

The future of veterinary public health surveillance lies in the development of truly integrated surveillance systems (multi country, multispecies, epidemiological metadata combined with sequence data) with sufficient analytical and reporting/early-warning capacity. Our ability to detect connections and aberrations within such systems is becoming crucial to our capacity in making sound decisions to improve health (18, 19). Data visualization can make an important contribution to this by providing an overview of the data; allowing quick identification of clusters, trends, gaps, or outliers; and enable a user to visually locate relationships and interactions in an easier way than with metadata tables for example (20). The wider uptake of timely reporting and interactive visualization tools in the context of animal and food safety, such as the ones we conceptualized for the FSVO, could play a valuable role to (a) improve early-warning capability through the timely identification of irregularities in surveillance data and (b) once a threat is verified, to better communication and plain reporting of said events to decision makers and relevant stakeholders in a timely manner.

## Author Contributions

UM and FV critically reviewed the paper for important intellectual content and made a lead contribution to the conception, design, analysis, and interpretation of the work. FW, DH, and MR critically reviewed the paper for important intellectual content and made a contribution to the conception, design, analysis, and interpretation of the work. PM drafted the paper and made contributions to the design, analysis, and interpretation of the work.

## Conflict of Interest Statement

The authors declare that the research was conducted in the absence of any commercial or financial relationships that could be construed as a potential conflict of interest.

## Footnotes

^1^https://www.equinella.ch/home/

^2^https://typo3.org/

^3^http://www.ted.com/talks/hans_rosling_shows_the_best_stats_you_ve_ever_seen
